# CD71 expressing circulating neutrophils serve as a novel prognostic biomarker for metastatic spread and reduced outcome in pancreatic ductal adenocarcinoma patients

**DOI:** 10.1038/s41598-024-70916-3

**Published:** 2024-09-10

**Authors:** Frederik J. Hansen, Anke Mittelstädt, Finn-Niklas Clausen, Samuel Knoedler, Leonard Knoedler, Sebastian Klöckner, Isabelle Kuchenreuther, Johanne Mazurie, Lisa-Sophie Arnold, Anna Anthuber, Anne Jacobsen, Susanne Merkel, Nadine Weisel, Bettina Klösch, Alara Karabiber, Irem Tacyildiz, Franziska Czubayko, Helena Reitberger, Amr El Gendy, Maximilian Brunner, Christian Krautz, Kerstin Wolff, Sidonia Mihai, Clemens Neufert, Jürgen Siebler, Robert Grützmann, Georg F. Weber, Paul David

**Affiliations:** 1 Department of General and Visceral Surgery, Friedrich-Alexander-University, Krankenhausstraße 12, 91054 Erlangen, Germany; 2 Institute of Regenerative Biology and Medicine, Helmholtz Center Munich, Ingolstädter Landtsraße 1, 85764 Neuherberg, Germany; 3 Division of Genetic Immunotherapy (LIT), University of Regensburg, Franz-Josef-Strauß-Allee 11, 93053 Regensburg, Germany; 4 Deutsches Zentrum Immuntherapie (DZI), Friedrich-Alexander-Universität Erlangen-Nürnberg and Universitätsklinikum Erlangen, Erlangen, Germany; 5 Bavarian Cancer Research Center (BZKF), Erlangen, Germany; 6 First Department of Medicine, Universitätsklinikum Erlangen, Friedrich-Alexander-Universität Erlangen-Nürnberg, Erlangen, Germany; 7 Zentrallabor im Universitätsklinikum, Universitätsklinikum Erlangen, Friedrich-Alexander-Universität Erlangen-Nürnberg, Krankenhausstr. 12, Erlangen, Germany

**Keywords:** Pancreatic ductal adenocarcinoma, Neutrophils, CD71, Transferrin receptor 1, Prognostic biomarker, Metastatic spread, Tumour immunology, Pancreatic cancer, Tumour biomarkers

## Abstract

Pancreatic ductal adenocarcinoma (PDAC) is one of the most lethal malignancies, presenting a persisting global health burden. Neutrophils have a double-edged role in tumor progression exhibiting both pro-tumor and anti-tumor functions. CD71, also known as transferrin receptor 1, performs a critical role in cellular iron uptake and is highly expressed on proliferating cells, and especially on activated immune cells. CD71 is known to be elevated in various types of solid cancers and is associated with poor prognosis, however, the expression of CD71 on neutrophils in PDAC and its potential clinical impact is still unknown. Therefore, we analyzed CD71 on circulating neutrophils in PDAC and clinical control patients and found a significant increased expression in PDAC patients. High expression of CD71 on neutrophils in PDAC patients was associated with reduced outcome compared to low expression. CD71 on neutrophils correlated positively with the levels of proinflammatory cytokines IL-6, IFN-γ, and growth factor ligands CD40-L, and BAFF in plasma of PDAC patients. Finally, we have demonstrated that high expression of CD71 on neutrophils was also associated with an increased expression of CD39 and CD25 on circulating T-cells. Based on our findings, we hypothesize that CD71 on neutrophils is associated with tumor progression in PDAC. Further studies are required to investigate the distinct functionality of CD71 expressing neutrophils and their potential clinical application.

## Introduction

Pancreatic ductal adenocarcinoma (PDAC) remains a persisting public health burden with ~ 500,000 new cases diagnosed globally in 2022^1,2^. While the five-year survival rate of PDAC is still dismal (~ 10%), its incidence is on the rise. By 2030, it is projected to become the second leading cause of cancer-related deaths in the U.S.^3,4^. These numbers underscore the urgent need for novel treatments including surgical and non-surgical approaches. However, the late diagnosis, the immunosuppressive tumor microenvironment, and the aggressive tumor growth persist as therapeutic hurdles diminishing the effects of conventional chemotherapy and immune-modulating treatments^5–7^.

The double-edged role of neutrophils in tumor progression has been subject to recent studies^8^. Many studies have shown that neutrophils could exhibit both pro-tumor and anti-tumor functions depending on their phenotype and the tumor microenvironment, among other parameters^9^. For instance, they release cytokines like IL-6 and TNF-α, or vascular endothelial growth factor (VEGF) and matrix metalloproteinases (MMP) that have been involved in cancer cell proliferation and tumor metastasis^10–12^. Vice versa, neutrophils can directly attack tumor cells secreting reactive oxygen species (ROS), elastase, or cathepsin G, which can induce apoptosis in cancer cells and limit tumor growth^13–16^.

CD71 is a cell surface glycoprotein that is involved in iron homeostasis, primarily by facilitating the intracellular uptake of iron-bound transferrin^17^. Within this context, cells that are actively dividing, especially immune cells that have been activated, demonstrate notably elevated levels of CD71^18^. This protein has been shown to play a crucial role in PDAC. For example, elevated gene expression of CD71 on pancreatic cancer cells have been correlated with larger tumor sizes, advanced PDAC stages, and an overall poorer prognosis^19,20^. However, few studies have focused on the expression of CD71 on immune cells.

Recently, the significance of CD71 on neutrophils has accumulated scientific interest with studies indicating the increased presence of CD71^+^ neutrophils in lung cancer and melanoma patients compared to healthy individuals^21^. Nevertheless, the significance of this cell population in PDAC and its possible implications in clinical settings are not well comprehended. Ultimately, the lack of research work may result in pioneering therapy potential and non-optimal patient care.

To bridge this knowledge gap, we aimed to investigate the expression of CD71 on neutrophils in PDAC patients and assess their clinical significance. Furthermore, we correlated the expression of CD71 with cytokines in plasma and pro-tumoral markers on circulating T cells.

## Materials and methods

### Patient samples

The present study was performed in accordance with the Declaration of Helsinki. Patients aged 18 years or older with a postoperative histopathological diagnosis of PDAC who underwent elective surgery at the Department of Surgery of the University Hospital Erlangen between 2020 and 2023 were eligible to be enrolled in the present study. In addition, patients who were undergoing treatment in the oncology outpatient clinic of Medicine 1 at University Hospital Erlangen were also included. Peripheral blood was collected from 21 control and 70 PDAC patients. The control group consists of patients who have neither cancer nor acute infection. Their characteristics are shown in Table 1.

**Table 1:** Characteristic features of the clinical control study cohort (n = 21).

	Clinical control patients
Number	21
Mean Age (in years [range])	63 (38–84)
Sex (Male:Female)	10:11
Performed surgery	
Cholecystectomy	8
Hernia repair surgery	3
Pancreatectomy for chronic pancreatitis (inflammation-free interval)	10

The clinic-pathological parameters of the PDAC study cohort are listed in Table 2. The TNM classification was applied according to the most recent edition of the Union of International Cancer Control (UICC)^22^.

**Table 2:** Characteristic features of the PDAC study cohort (n = 70).

	PDAC patients
Number	70
Mean Age (in years [range])	67 (45–92)
Sex (Male:Female)	36:34
pT-category	
1	6
2	32
3	12
4	3
Unknown/Inoperable	17
pN-category	
pN0	29
pN +	24
Unknown/Inoperable	17
Perineural invasion	
Pn0	27
Pn +	26
Unknown/Inoperable	17
Venous invasion	
V0	45
V +	8
Unknown/Inoperable	17
Lymphatic invasion	
L0	42
L +	11
Unknown/Inoperable	17
R-status	
R0	47
R1,2,X	23
Grading	
G2	28
G3	21
Unknown	21
Distant Metastasis	
No	50
Yes	20
UICC stage	
I	27
II	15
III	8
IV	20

### Sample preparation

The blood samples were obtained preoperative in 7.5 mL EDTA tubes (Cat-No. 04.1921.001, Sarstedt, Nürnbrecht, Germany). In the first step, plasma was separated at a centrifugation of 350 × g for 10 min without brakes. Subsequently, 30 mL of erythrocyte lysis buffer (Cat-No. 555899, BD Bioscience, Franklin Lakes, NJ, USA) was added to the cells followed by 15 min incubation at room temperature. Then, the cells were centrifuged at 350 × g for 5 min and resuspended in 50 mL PBS (Cat-No. 14190169, Gibco, Waltham, MA, USA). Afterwards, leukocytes were counted with trypan blue under the microscope to assess the absolute cell numbers. In the last step, the cell suspensions were centrifuged at 350 g for 5 min at 4 °C , and the cell concentration was adjusted to maximum 1 million cells per 100 μl in FACS Buffer [PBS containing 1% FBS (Cat-No. A3160802. Gibco, Waltham, MA, USA), 0.5% BSA (Cat-No. A2153, Sigma-Aldrich, St. Louis, MO, USA), and 2 mM EDTA (Cat-No. AM9260G, Invitrogen, Waltham, MA, USA)] for flow cytometric analyses.

### Flow cytometry

The following antibodies were used in the present study: anti-CD15-PE (Cat-No. 562371, BD Biosciences, Franklin Lakes, NJ, USA), anti-CD71-BV650 (Cat-No. 743307, BD Biosciences, Franklin Lakes, NJ, USA), anti-CD3-BUV737 (Cat-No. 564308, BD Biosciences, Franklin Lakes, NJ, USA), anti-CD45-BV786 (Cat-No. 563716, BD Biosciences, Franklin Lakes, NJ, USA), anti-HLADR-BUV395 (Cat-No. 565972, BD Biosciences, Franklin Lakes, NJ, USA), anti-CD80-PE (Cat-No. 560925, BD Biosciences, Franklin Lakes, NJ, USA), anti-CD69-BV421 (Cat-No. 562883, BD Biosciences, Franklin Lakes, NJ, USA), anti-CD86-BV510 (Cat-No. 563460, BD Biosciences, Franklin Lakes, NJ, USA), anti-PDL-1-BV650 (Cat-No. 563740, BD Biosciences, Franklin Lakes, NJ, USA), anti-CD25-BUV711 (Cat-No. 740776, BD Biosciences, Franklin Lakes, NJ, USA), anti-CD39-BUV737 (Cat-No. 564726, BD Biosciences, Franklin Lakes, NJ, USA), anti-CD42a-BV650 (Cat-No. 743728, BD Biosciences, Franklin Lakes, NJ, USA), anti-CD14-BUV737 (Cat-No. 612763, BD Biosciences, Franklin Lakes, NJ, USA), anti-CD16-FITC (Cat-No. 406555, BD Biosciences, Franklin Lakes, NJ, USA). Data were acquired using a Celesta (BD Biosciences, Franklin Lakes, NJ, USA) flow cytometer using the BD FACSDiVa™ software v8.0.1.1 and analyzed with FlowJo 10.9.0 (FlowJo LLC, Ashland, OR, USA).

### Multiplex cytokines analysis

As described above, plasma was separated from peripheral blood at 350 g for 10 min without brakes. Then, serum levels of cytokines were measured using LEGENDPlexTM bead-based immunoassays (Cat-No. 740527, Biolegend, San Diego, CA, USA) according to the manufacturer’s instructions. IL-6, IFN-γ, TNF-α, IL-10, CD40-L and BAFF were simultaneously quantified. Data acquisition was performed on BD FACS Fortessa flow cytometer (BD Biosciences, Franklin Lakes, NJ, USA) and analyzed with the LEGENDPlexTM Data Analysis Software (Biolegend, San Diego, CA, USA). The results are shown as mean fluorescence intensity (MFI).

### Measurement of ferritin, transferrin, hemoglobin and platelets

Ferritin (OSR 61,203) and transferrin (OSR 6152) were measured using immunoturbidimetry (Beckman Coulter DxC 700 AU). Platelets and hemoglobin were using Sysmex XN-1000 according to the manufacturer’s instructions.

### Human quantikine myeloperoxidase immunoassay ELISA

The Quantikine™ ELISA Human Myeloperoxidase Immunoassay (R&D Systems; DMYE00B) was performed according to the manufacturer’s instructions. In brief a tenfold dilution of plasma samples from PDAC and control patients was added to the provided 96 well microplate pre-coated with a monoclonal antibody specific for human MPO for two hours at room temperature followed by 4 °C in the refrigerator overnight. After washing an enzyme-linked polyclonal antibody specific for human MPO was added. After another washing step substrate solution was added and blue color developed proportional to the initial amount of MPO in the samples. The development was stopped after 30 min and intensity measured using a plate reader SpectraMax M3 by Molecular Devices.

### Statistical analysis

Statistics were determined using GraphPad Prism (V9.4.0 for macOS, GraphPad Software, La Jolla, CA, USA). For comparison of measurement data between 2 groups, an unpaired t-test was applied. For counting data, the Pearson Chi-square test was applied. Survival data was analyzed using the Log-rank (Mantel-Cox) test. A simple linear regression was performed for the correlation. The regression line was plotted and, in addition to the p-value, the coefficient of determination R2, which represents the measure of quality of the linear regression, was also provided. Odds ratio with 95% Confidence Interval (CI) was determined using IBM SPSS Statistics 29.0.1 software (Armonk, NY, USA). Due to the low number of patients, no outliers were excluded. Differences were considered statistically significant at *p* < 0.05. All p values are two-tailed.

### Institutional review board statement

The study was conducted in accordance with the Declaration of Helsinki and approved by the Institutional Review Board of the University Hospital Erlangen (Nr. 180_19 B, 14.06.2019).

## Results

### Increased expression of CD71 on neutrophils in peripheral blood associates with reduced survival in PDAC patients

**Fig. 1:**
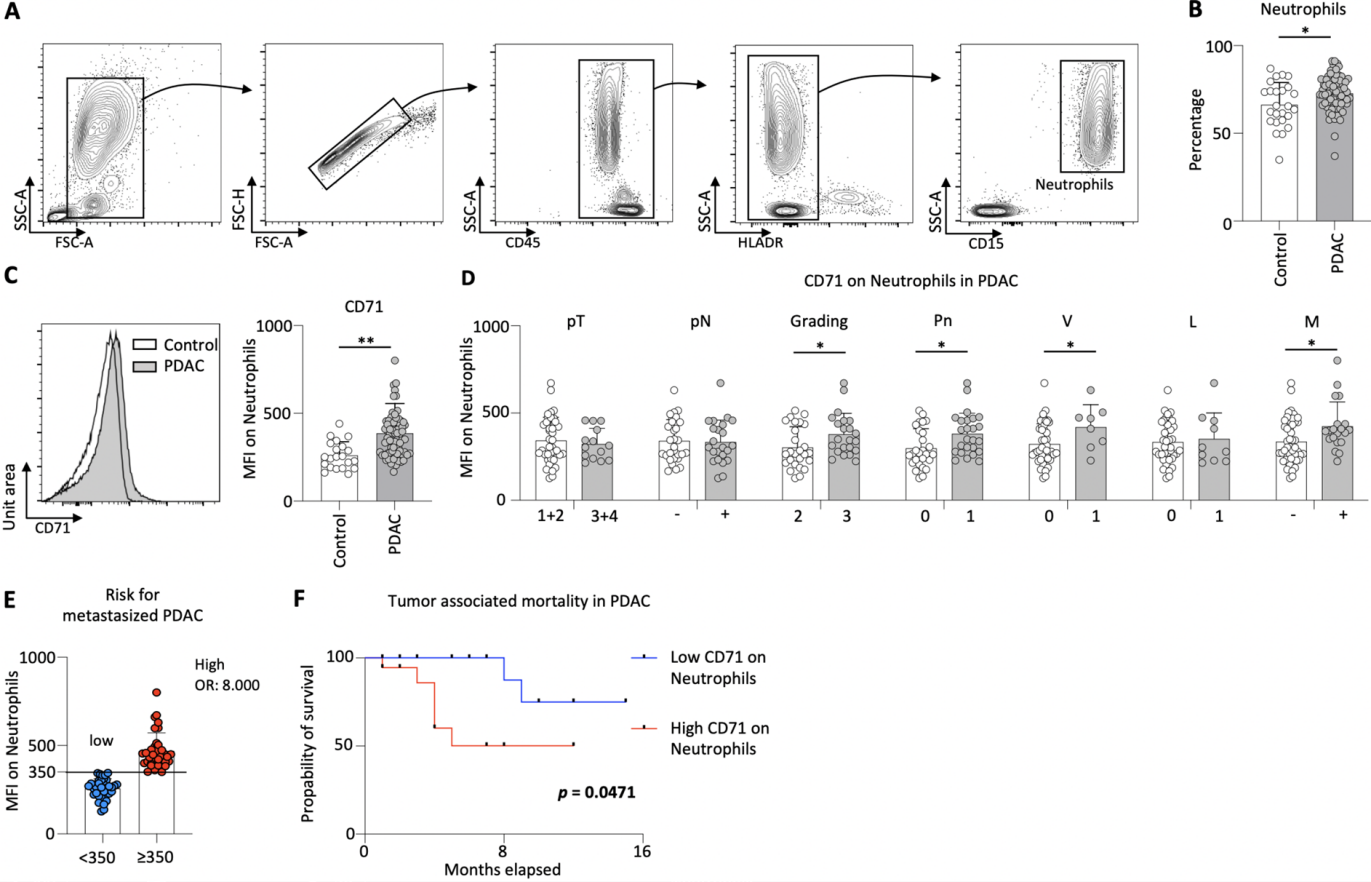
PDAC patients showed increased expression of CD71 on neutrophils. Gating strategy to characterize circulating neutrophils (**A**); percentage of circulating neutrophils in clinical control (n = 21) and PDAC patients (n = 70) (**B**); histogram and bar plot denoting the expression of CD71 on circulating neutrophils in clinical control and PDAC patients (**C**); expression of CD71 on circulating neutrophils in PDAC patients correlated to the pT category (pT), pN category (pN), Grading, perineural invasion (Pn), Venous invasion (V), lymphatic invasion (L) and distant metastasis (M) (**D**); MFI of CD71 on neutrophils (350 identified by minimal *p*-value approach) defining the risk for metastasized PDAC (E); survival analysis for PDAC patients with high (≥ 350) and low (< 350) CD71 expression on circulating neutrophils (F); *p < 0.05; **p < 0.01.

Myeloid cells play a crucial role in the progression of pancreatic cancer^23^. Previously, we have shown the impact of circulating monocytes in predicting the clinical outcome of PDAC patients^24^. Here, we investigated the frequencies of circulating neutrophils in peripheral blood of clinical control and PDAC patients. The gating strategy to characterize neutrophils in circulation is shown in Fig. 1A. PDAC patients (n = 70) showed significantly higher frequencies of neutrophils in comparison to clinical control patients (n = 21) (Fig. 1B).

CD71 is responsible for the uptake of iron into the cell and is expressed on cells with high proliferation. Particularly on T cells, CD71 is well established as an activation marker^25^, but not much is known about it on neutrophils. Recent report in lung cancer showed their role in neutrophils^21^. Therefore, we determined the expression (MFI) of CD71 on neutrophils and found that PDAC patients had a significant higher expression compared to clinical control patients (Fig. 1C).

We then investigated the impact of the expression of CD71 on neutrophils on the clinic-pathological characteristics in PDAC patients. To this end, we grouped the study cohort according to the tumor size and did not find any difference between patients with localized (pT1 and pT2, n = 38) and locally advanced (pT3 and pT4, n = 15) tumors with respect to the MFI of CD71 on neutrophils. Concerning the status of lymph node involvement, no difference between patients with positive (pN + , n = 24) and negative lymph nodes (pN-, n = 29) was detected. The analysis of the Grading revealed a significant increase in G3 (n = 21) comparing to G2 (n = 28) tumors. Regarding perineural invasion of the tumor, significant higher expression of CD71 was observed in patients with perineural invasion (Pn1, n = 26) in comparison to patients without perineural invasion (Pn0, n = 27). Patients with venous invasion (V1, n = 8) showed a higher expression of CD71 on circulating neutrophils than patients without venous invasion (V0, n = 45). In contrast, the analysis of lymphatic invasion (L0, n = 42 vs. L1, n = 11), no difference was observed. Patients with distant metastasis (M + , n = 20) showed a notable increase in the expression of CD71 on circulating neutrophils than to those that were localized. (M-, n = 50) (Fig. 1D).

As higher expression of CD71 on neutrophils was associated with metastatic spread, the role of CD71 expressing neutrophils as prognostic biomarker for disease severity was investigated. To identify the CD71 expression level that correlates with the highest probability for metastasized PDAC occurrence, a minimal p-value approach (Table 3) was performed. Patients with an expression of CD71 on neutrophils above 350 were more likely to suffer from metastatic spread. More precisely, patients with a CD71 expression on neutrophils greater then 350 had an eightfold increased risk for metastatic spread compared to patients with an expression below 350 (OR 8.000, 95% CI 2.054–31.159) (Fig. 1E).

**Table 3:** Determining the cutoff threshold of the expression of CD71 on neutrophils based on the metastatic spread using the two-tailed minimal p-value approach (chi-square test; n = 70).

MFI CD71 (%)	p-value (chi-sqare test)	CD71 Expression	N	Probability of metastasis (%)
200	0.14	Low	5	0
		High	65	44.4
250	0.05	Low	14	7.1
		High	56	34.0
300	< 0.01	Low	26	3.8
		High	44	43.2
**350**	** < 0.01**	**Low**	**35**	**8.6**
		**High**	**35**	**48.6**
400	< 0.01	Low	42	16.7
		High	28	46.4
450	0.19	Low	53	24.5
		High	17	41.2
500	0.26	Low	61	26.2
		High	9	44.4

The optimal cutoff value with the lowest *p*-value and the highest probability of metastatic spread is highlighted in bold.

To assess the reliability of the recently implemented prognostic biomarker, an analysis of patient survival was conducted, stratified by the CD71 expression on circulating neutrophils. PDAC patients with a low expression (< 350; n = 35) exhibited significantly higher survival rates compared to those with high expressing CD71^+^ neutrophils (≥ 350; n = 35) (Fig. 1F). Table 4 provides details on the characteristic features of the study population, categorized based on CD71 expression on neutrophils.

**Table 4:** Characteristic features of the study cohort grouped according to the mean expression of CD71 on neutrophils into low (n = 35; < 350) and high (n = 35; ≥ 350). Significant values are in bold.

Expression of CD71	Low	High	p-value
Number	35	35	
Mean Age (in years [range])	67 (45–87)	68 (48–92)	0.8946
Sex			
Male	20	16	0.34
Female	15	19	
pT-category			
1	3	3	0.88
2	18	14	
3	8	4	
4	2	1	
Unknown/Inoperable	4	13	
pN-category			
pN0	17	12	0.98
pN +	14	10	
Unknown/Inoperable	4	13	
Venous invasion			
V0	28	17	0.19
V +	3	5	
Unknown/Inoperable	4	13	
Lymphatic invasion			
L0	25	17	0.77
L +	6	5	
Unknown/Inoperable	4	13	
Perineural invasion			
Pn0	19	8	0.07
Pn +	12	14	
Unknown/Inoperable	4	13	
R-status			
R0	27	20	0.07
R1,2,X	8	15	
Grading			
G2	19	9	0.27
G3	11	10	
Unknown	5	16	
Distant Metastasis			
No	30	20	** < 0.01**
Yes	5	15	
UICC stage			
I	16	11	0.07
II	9	6	
III	5	3	
IV	5	15	

We also analyzed the expression of CD71 on monocytes and T cells (Supplementary Fig. 1) to assess whether the results described above also apply to these immune cells. The analysis of clinical control and PDAC patients and their clinicopathological characteristics showed more significant results for neutrophils compared to monocytes or T cells.

### CD71 correlates with other activation markers on neutrophils in PDAC patients

Next, we correlated CD71 expressing neutrophils with other activation markers expressed on neutrophils in PDAC patients to assess whether CD71 could also serve as an activation marker on neutrophils. For this purpose, we analyzed activation markers that have not been well studied in neutrophils but are known to interact with T-cells.

**Fig. 2:**
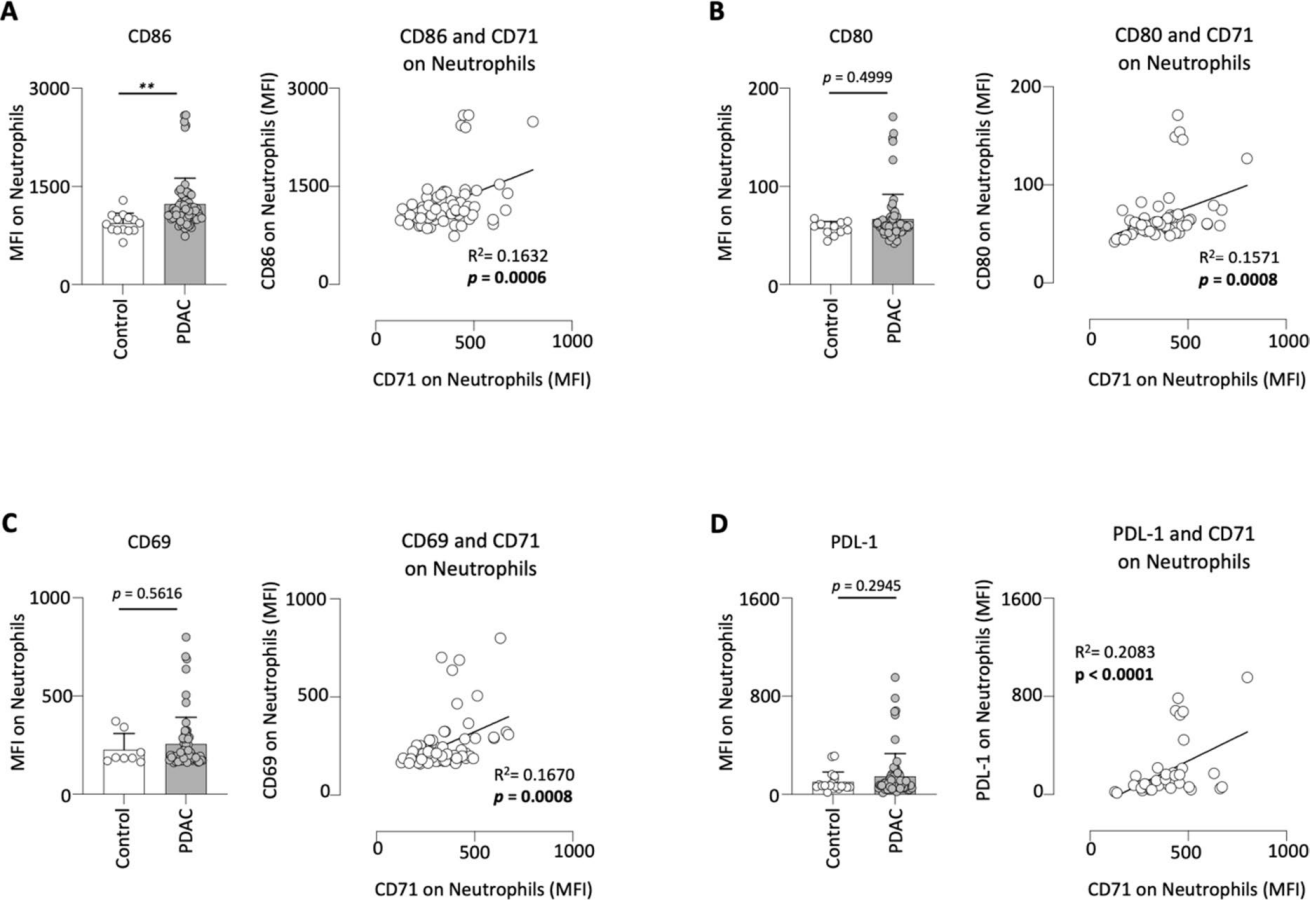
Expression of CD71 on neutrophils correlated positively with CD86, CD80 and CD69 on neutrophils in PDAC patients. Expression of CD86 on neutrophils in clinical control (n = 14) and PDAC patients and the correlation of CD86 with CD71 on neutrophils in PDAC patients (n = 70) (**A**); expression of CD80 on neutrophils in clinical control (n = 12) and PDAC patients and the correlation of CD80 with CD71 on neutrophils in PDAC patients (n = 70) (**B**); expression of CD69 on neutrophils in clinical control (n = 9) and PDAC patients and the correlation of CD69 with CD71 on neutrophils in PDAC patients (n = 70) (**C**); expression of PDL-1 on neutrophils in clinical control (n = 21) and PDAC patients and the correlation of PDL-1 with CD71 on neutrophils in PDAC patients (n = 70) (**D**); **p < 0.01.

First, we analyzed CD86 and found that PDAC patients had significantly higher expression compared to clinical controls. Furthermore, the expression of CD86 correlated significantly with CD71 on circulating neutrophils (Fig. 2A). Analysis of CD80 (Fig. 2B) and CD69 (Fig. 2C) showed no difference between clinical control and PDAC patients, but both activation markers correlated with the MFI of CD71 on neutrophils. Finally, PDAC patients showed a tendency to increased PDL-1 expression on neutrophils compared to control patients. A significant correlation with the expression of CD71 was also observed (Fig. 2D). We also analyzed the concentration of myeloperoxidase (MPO) in the plasma. This enzyme is produced by neutrophils upon activation. PDAC patients showed a tendency towards increased levels compared to clinical controls and the correlation with CD71 on neutrophils showed no correlation (Supplementary Fig. 2).

In conclusion, we could show that CD71 correlates with several activation markers and thus could itself be considered an activation marker for neutrophils, among others.

### Expression of CD71 on neutrophils correlates with the activation of platelets in PDAC patients

**Fig. 3:**
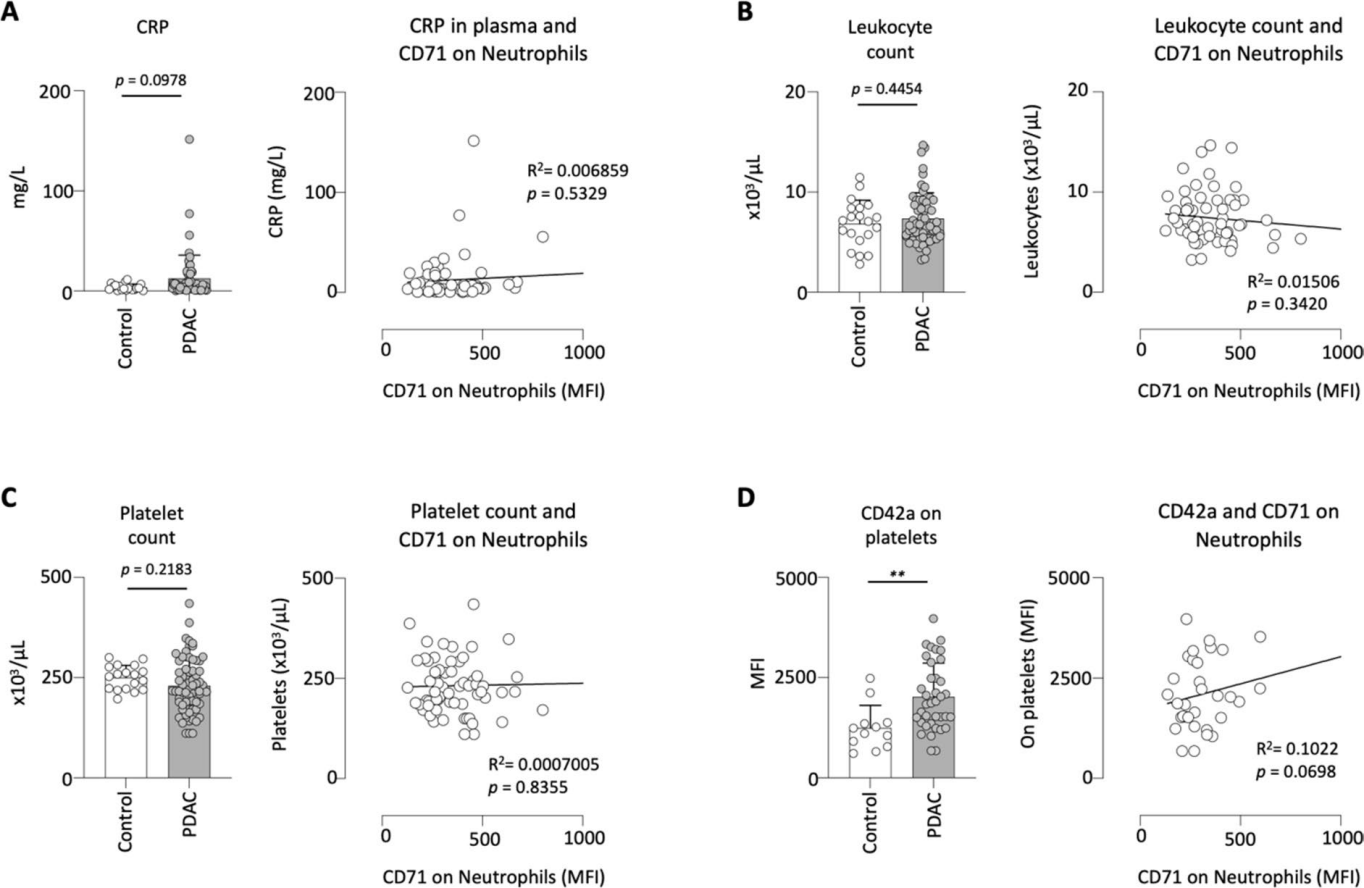
Expression of CD71 on neutrophils correlates with the activation of platelets in PDAC patients. CRP values in the plasma of clinical control (n = 21) and PDAC patients (n = 70). CRP values in plasma correlated to the expression of CD71 on neutrophils in PDAC patients (**A**); leukocyte count in the blood of clinical control (n = 21) and PDAC patients (n = 70). Leukocyte count in blood correlated to the expression of CD71 on neutrophils in PDAC patients (**B**); platelet count in the blood of clinical control (n = 21) and PDAC patients (n = 70). Platelet count in blood correlated to the expression of CD71 on neutrophils in PDAC patients (**C**); Expression of the activationmarker CD42a on platelets in clinical control (n = 14) and PDAC patients (n = 45) and the correlation of CD42a on platelets with CD71 on neutrophils in PDAC patients (**D**); **p < 0.01.

Next, we correlated the expression of CD71 on neutrophils with important clinical blood parameters. First, we analyzed values of systemic inflammation. We found a tendency to increased C-reactive Protein (CRP) levels in PDAC patients compared to clinical controls (*p* = 0.0978). The correlation of this unspecific inflammation marker with the expression of CD71 on circulating neutrophils was not significant (Fig. 3A).

Afterwards, an analysis of the leukocyte count in the blood was conducted. Here, no significant difference was observed between the clinical control group and PDAC patients (*p* = 0.4454). Additionally, there was no correlation found between the leukocyte count in the blood and the CD71 expression on circulating neutrophils in PDAC patients (Fig. 3B).

Since PDAC patients have increased platelet activity and therefore an increased risk of thromboembolism, we investigated the number of platelets in the blood. We found only a slight tendency towards a lower platelet count in PDAC patients compared to clinical controls (*p* = 2183). The subsequent correlation with CD71 on circulating neutrophils showed no significance (Fig. 3C). However, as the number of platelets only provides limited information about the activation status of the platelets, we also analyzed CD42a on platelets. CD42a also known as glycoprotein IX forms a complex that functions as a receptor for von Willebrand factor^26^. We found that PDAC patients had an increased expression of CD42a on platelets compared to clinical controls. The correlation with CD71 on neutrophils also showed a tendency towards a positive correlation (*p* = 0.0698) (Fig. 3D).

### Expression of CD71 on neutrophils is linked to the ferritin levels in PDAC patients

**Fig. 4:**
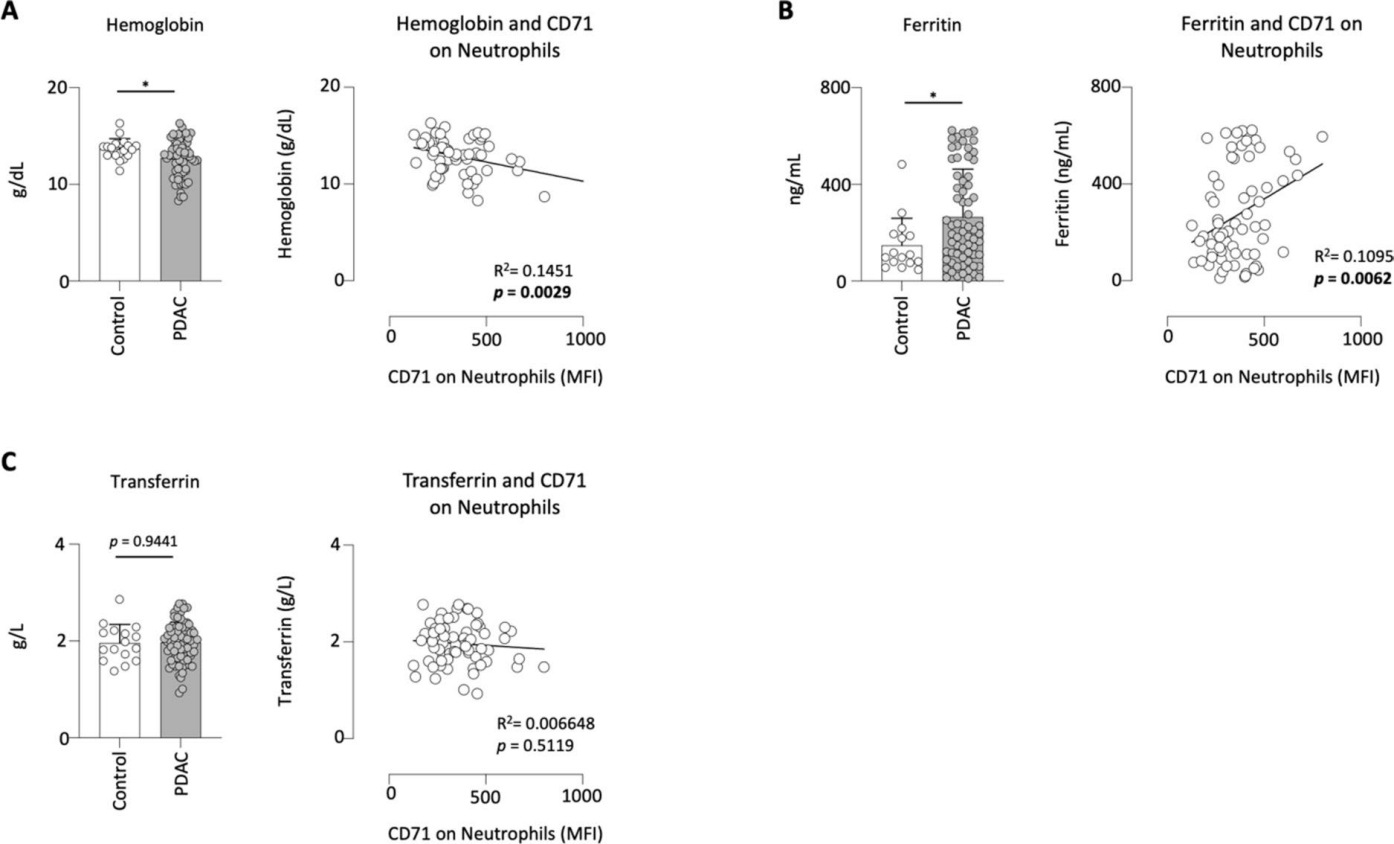
Expression of CD71 on neutrophils is linked to the ferritin levels in PDAC patients. Hemoglobin levels of clinical control (n = 21) and PDAC patients (n = 70). Hemoglobin levels correlated to the expression of CD71 on neutrophils in PDAC patients (**A**); ferritin levels of clinical control (n = 16) and PDAC patients (n = 70). Ferritin levels correlated to the expression of CD71 on neutrophils in PDAC patients (**B**); transferrin levels in clinical control (n = 16) and PDAC patients (n = 70) and the correlation of ferritin with CD71 on neutrophils in PDAC patients (**C**); *p < 0.05.

Since CD71 is the receptor for transferrin, we analyzed the iron status of our PDAC cohort to investigate a possible correlation with the expression of CD71 on neutrophils. A disorder of the iron balance is often characterized by a low hemoglobin value. Therefore, we analyzed this value and found that PDAC patients had significantly lower hemoglobin values compared to clinical controls. Subsequent correlation with CD71 on neutrophils showed that patients with a high expression of CD71 had lower hemoglobin levels (Fig. 4A).

This was followed by an analysis of ferritin. Ferritin is a protein complex that stores and releases iron in the body, playing a critical role in regulating iron homeostasis. PDAC patients showed significantly higher levels of ferritin than clinical control patients. Interestingly, the ferritin level correlated markedly with the expression of CD71 on neutrophils (Fig. 4B).

Transferrin is a transport protein involved in iron homeostasis, binding ferric ions and facilitating their transport through the bloodstream. No difference was observed between control and PDAC patients. The correlation of transferrin levels with CD71 expression on circulating neutrophils also revealed no significance (Fig. 4C).

### Expression of CD71 on neutrophils is signifacntly linked to increased levels of IL-6, IFN-γ, CD40-L and BAFF in the plasma of PDAC patients

Neutrophils have a variety of functions during cancer disease^27^. Therefore, the aim was to determine whether the expression of CD71 on neutrophils is associated with important proinflammatory and anti-inflammatory cytokines, as these might also play an important role in tumor progression.

**Fig. 5:**
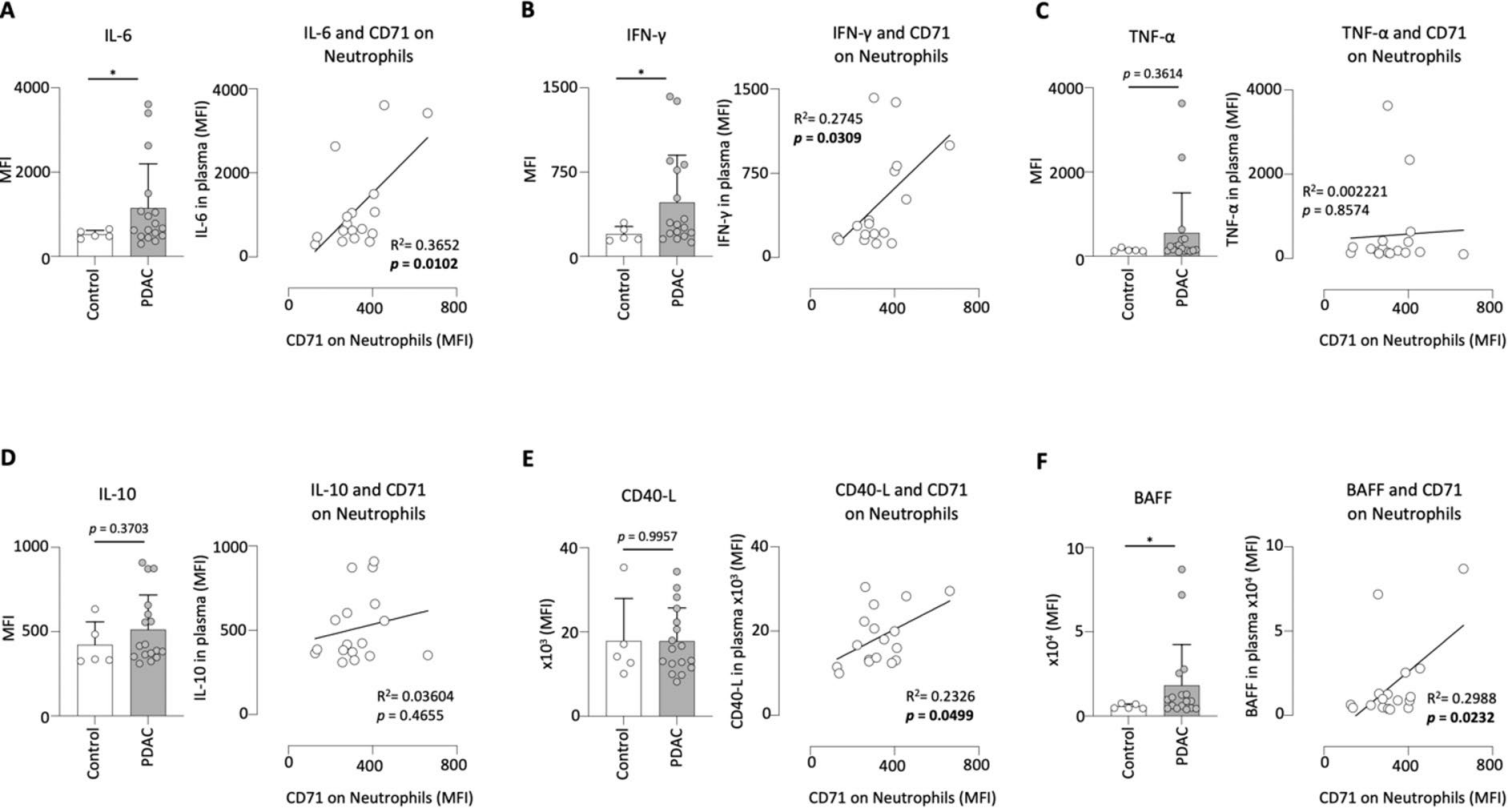
Expression of CD71 on neutrophils correlated positively with the IL-6, IFN-γ, CD40-L and BAFF plasma levels in PDAC patients. Levels of IL-6 (**A**), IFN-γ (**B**), TNF-α (**C**), IL-10 (**D**), CD40-L (**E**) and B-cell activating factor (BAFF) (F) in the plasma of clinical control (n = 5) and PDAC patients (n = 17) and the correlation to the MFI of CD71 on circulating neutrophils; *p < 0.05.

First, we analyzed the strongly pro-inflammatory cytokine IL-6 and found increased levels in PDAC patients compared to clinical controls. These levels also correlated significantly with the expression of CD71 on neutrophils (Fig. 5A). IFN-γ was also elevated in PDAC patients and correlated with CD71 expression on circulating neutrophils (Fig. 5B). Regarding the levels of TNF-α (Fig. 5C) and IL-10 (Fig. 5D), only a tendential increase was observed in PDAC patients. There was no significant correlation between both cytokines and CD71 expression on neutrophils. The analysis of the CD40 ligand (CD40-L) showed no difference between clinical controls and PDAC patients, however, levels correlated with the expression of CD71 on circulating neutrophils (Fig. 5E). Finally, we demonstrated that PDAC patients had significantly higher levels of B cell activating factor (BAFF) and these also correlated with MFI of CD71 on neutrophils (Fig. 5F).

### CD71 on neutrophils correlated positivley with the activation status of T-cells in the blood of PDAC patients

**Fig. 6:**
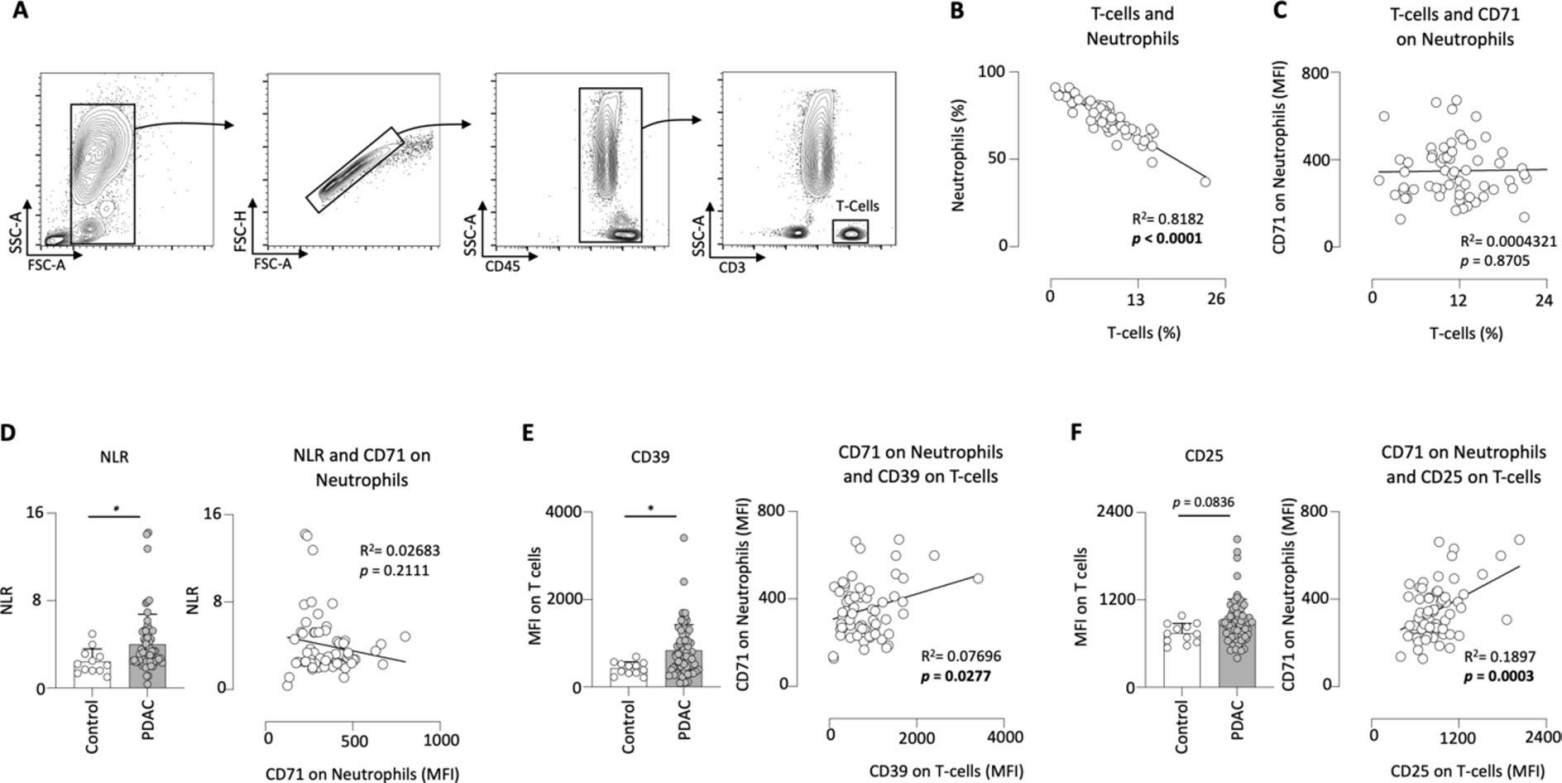
The frequencies of neutrophils correlated negatively with the frequencies of T-cells in PDAC patients. Gating strategy for circulating T cells (**A**); percentage of neutrophils correlated to the percentage of T-cells in the blood of PDAC patients (n = 70) (**B**); expression of CD71 on neutrophils correlated to the percentage of T-cells (**C**); the prognostic Neutrophil-to-Lymphocytes-Ratio (NLR) in clinical control (n = 12) and PDAC patients and the correlation of NLR with CD71 on neutrophils in PDAC patients (n = 70) (**D**); expression of CD39 on T cells in clinical control (n = 12) and PDAC patients and the correlation of CD39 on T cells with CD71 on neutrophils in PDAC patients (n = 70) (**E**); expression of CD25 on T cells in clinical control (n = 12) and PDAC patients and the correlation of CD25 on T cells with CD71 on neutrophils in PDAC patients (n = 70) (**F**); *p < 0.05.

T-cells are one of the main constituent of the adaptive immune system and hold a very crucial role in carcinogenesis and are already been utilized as targets for various experimental therapeutic approaches^28^. Therefore, we analyzed the frequencies of T-cells and the expression of activation markers on T-cells, which are suspected to promote tumor progression, and correlated these with the frequencies of neutrophils and the MFI of CD71 on neutrophils in PDAC patients. The gating strategy for identifying T-cells in the peripheral blood is shown in Fig. 6A.

We found that the frequencies of T-cells were negatively correlated with the frequencies of neutrophils in the blood of PDAC patients (Fig. 6B), while no correlation was observed between the percentage of T-cells and the expression of CD71 on neutrophils (Fig. 6C).

Since the frequencies of neutrophils correlated negatively with the frequencies of T cells, we calculated the neutrophil-to-lymphocyte ratio (NLR). This ratio has already been established as a prognostic marker for PDAC in other studies. It could be shown that an increased NLR was associated with poorer survival^29^. PDAC patients in our cohort showed an increased NLR compared to clinical controls. However, the correlation with CD71 on neutrophils was not significant, in fact a slight negative trend was observed (Fig. 6D).

Next, we correlated the MFI of CD71 on neutrophils with the expression of CD39 on T-cells. CD39 is an ectonucleotidase that converts ATP into immunosuppressive extracellular adenosine^30^. We could show that the expression of CD39 on T-cells positively correlated with the expression of CD71 on neutrophils in PDAC patients (Fig. 6E). This supports the possibility that CD71 expressing neutrophils has immunosuppressive role and might be involved in tumor progression.

Finally, the correlation between CD71 on neutrophils and CD25 on T-cells was determined. CD25 is the IL-2 receptor on T-cells and is upregulated upon activation^31,32^. We were able to show a positive correlation between the expression of CD25 on T-cells and CD71 on neutrophils (Fig. 6F).

## Discussion

In this prospective study, we were able to show that PDAC patients had a higher expression of CD71 on circulating neutrophils compared to clinical control patients. In addition, PDAC patients with a higher grading, perineural invasion, venous invasion and distant metastasis, among others, showed increased CD71 expression on circulating neutrophil, resulting in reduced outcome. Furthermore, CD71 correlated with the T-cell interacting activation markers CD86, CD80 and CD69 on neutrophils. In addition, a positive significant correlation was found between the expression of CD71 on neutrophils and the levels of proinflammatory cytokines Il-6, IFN-γ, CD40-L, and BAFF in plasma of PDAC patients. In conclusion, we have demonstrated that high expression of CD71 on neutrophils was also associated with the increased expression of pro-tumoral markers on T-cells.

Neutrophils, key players in innate immunity, exert a significant impact on tumor progression through direct mechanisms, such as facilitating cell proliferation and genetic instability, and indirect mechanisms, including suppressing anti-tumor immune responses and promoting metastatic spread^33^. A recent single-cell RNA-sequencing analysis identified a pro-tumoral subset of tumor associated neutrophils correlated with poor prognosis in PDAC^34^. Consistent with this, our study reveals elevated neutrophil frequencies in PDAC compared to clinical control patients.

CD71, also known as transferrin receptor 1, is widely expressed on various cell types and performs a critical role in cellular iron uptake through the interaction with transferrin^18,35–38^. Interestingly, CD71 correlated with ferritin levels of PDAC patients but not with transferrin levels, although CD71 is the receptor for transferrin. Ferritin is a protein complex that serves to store iron. It is also an acute-phase protein and is therefore elevated in cases of systemic inflammation. A study was able to show that the uptake of ferritin in PDAC cells is increased and thus strongly promotes tumor growth^39^. Therefore, it is consistent that the expression of CD71 on neutrophils is associated with poorer outcome, increased pro-inflammatory cytokines and increased ferritin levels. In contrast, transferrin as a transport protein for iron is decreased in systemic inflammation^40^. Therefore, this is consistent with our finding of absence of correlation with CD71 expression on neutrophils in PDAC patients. We were able to prove that increased expression of CD71 on neutrophils is associated with higher systemic inflammation.

Proliferating cells, and especially activated immune cells exhibit a particularly high expression of CD71^18^. Studies demonstrated increased expression of CD71 on cancer cells and an associated worsened prognosis in cancer diseases such as breast cancer^41^, esophageal squamous cell carcinoma^42^, and cervical cancer^43^. However, not many studies focused on the expression of CD71 on immune cells such as neutrophils, which are the most abundant myeloid cells in human blood^27^. To date, only in the blood of melanoma and lung cancer patients an increased expression of CD71 on neutrophils compared to control patients has been described^21^. Until now, the expression of CD71 on neutrophils and the possible clinical relevance in PDAC was not known. This knowledge gap could be partially filled with the present study.

During early stages of head and neck cancer, tumor associated neutrophils develop an antigen-presenting phenotype (CD80^+^ CD86^+^ PDL-1^-^) and stimulate an anti-tumoral T-cell response, whereas in a more advanced tumor stage this phenotype expresses PDL-1 and thus has an immunosuppressing effect and thereupon promotes tumor progression^44^. In this present study, we demonstrated that CD71 correlated significantly with CD80, CD86 and PDL-1 on neutrophils. This correlation could suggest that CD71 expression on neutrophils is more likely to be associated with the pro-tumoral phenotype of neutrophils. However, further studies are required to determine the exact phenotype of CD71 expressing neutrophils in PDAC.

Neutrophils produce various cytokines influencing both tumor progression and anti-tumoral immune responses^45^. This includes IL-6, which is known to promote oncogenesis, angiogenesis through the induction of VEGF, and metastasis in PDAC^46–49^. In the present study, a positive correlation was found between IL-6 levels in plasma and the expression of CD71 on neutrophils, supporting our hypothesis that the expression of CD71 is associated with tumor progression in PDAC. In addition, we demonstrated a positive correlation with IFN-γ, a major driver in promoting innate and adaptive immune responses^50^. The role of this cytokine during the course of cancer is quite controversial. On the one hand, IFN-γ plays a central role in the recognition and elimination of cancer cells, on the other hand, cancer cells can take advantage of IFN-γ as an inducer of mediators inhibiting anti-tumoral immune response^51^. Therefore, further studies are needed to assess the potential impact of CD71 expressing neutrophils on the functionality of IFN-γ in PDAC. Soluble CD40-L is predominantly secreted by activated T cells and platelets^52^. The CD40-CD40L interaction produces many angiogenesis-associated factors, such as VEGF, hence a high serum CD40-L level is associated with a worse outcome in PDAC patients^53^. Therefore, the positive correlation of CD71 on neutrophils and the level of CD40-L in the serum of PDAC patients supports our possible hypothesis that CD71 expressing neutrophils may be associated with tumor progression. The B cell-activating factor (BAFF) belongs to the tumor necrosis factor and was found to be involved in tumor cell proliferation, survival and invasion^54^. In PDAC, higher BAFF expression is associated with disease severity and metastasis^55^. As BAFF serum levels correlated positively with CD71 expression on neutrophils, this is consistent with our possible hypothesis that CD71 on neutrophils is associated with tumor progression.

T-cells play a crucial role in the elimination of cancer cells and are therefore already successfully used therapeutically e.g., in CAR-T cell therapy. However, not all patients benefit, as T cells can become dysfunctional shaped by multifaceted suppressive signals^28^. The expression of CD71 on neutrophils correlated with CD86, CD80 and PDL-1, suggesting that CD71-expressing neutrophils may interact with T cells. CD39 is an ectonucleotidase that converts ATP into immunosuppressive extracellular adenosine, which leads to cancer immune evasion^56,57^. Several studies have demonstrated that antagonism of CD39 was able to restore antitumor immunity^58^. Interestingly, the expression of CD39 on T cells correlated with CD71 on neutrophils, suggesting that CD71-expressing neutrophils may be involved in T cell dysfunctionality in PDAC. Another fact supporting this hypothesis is that the expression of CD25 on T cells also positively correlated with the expression of CD71 on neutrophils. CD25 is the alpha-chain of the IL-2 receptor and is highly expressed in activated circulating immune cells and regulatory T-cells (Tregs), which are crucial for tumor immune evasion^59^. Currently, studies are underway to antagonize CD25 in cancer diseases^60^. Further studies are warranted to address the exact effect of CD71 expressing neutrophils on T cells and their subtypes in PDAC.

Limitations must be taken into consideration when analyzing the findings of the present study. Due to the relatively low number of patients, the statistical power is limited. Furthermore, since we did not analyze tumor infiltrating neutrophils and only performed correlations with pro-tumoral factors, we can only make assumptions about the role of CD71 neutrophils in tumor progression of PDAC. In addition, no ex vivo experiments were performed to precisely investigate the exact functionality such as cytokine production of CD71 expressing neutrophils. Therefore, it is crucial to conduct further studies to investigate the distinct functionality and their potential clinical application.

## Conclusions

The present study showed that a high expression of CD71 on circulating neutrophils in PDAC patients was associated with more aggressive tumor growth and an increased rate of metastasis and thus correlated with poorer survival. Further studies are needed to evaluate CD71 on neutrophils as a prognostic biomarker or as a potential therapeutic target in PDAC patients.

## Supplementary Information

Supplementary Information.

## Data Availability

For all data requests, please contact the corresponding author Georg F. Weber.

## Abbreviations

VEGF: Vascular endothelial growth factor
MMP: Matrix metalloproteinase
ROS: Reactive oxygen species
PDAC: Pancreatic Ductal Adenocarcinoma
MFI: Mean fluorescence intensity
pT: PT category
pN: PN category
Pn: Perineural invasion
V: Venous invasion
L: Lymphatic vessel invasion
R: Residual tumor classification
M: Metastasis
UICC: The Union of International Cancer Control
CRP: C-reactive protein
PDL-1: Programmed cell death 1 Ligand 1
TNF: Tumor-necrosis factor
IL: Interleukin
BAFF: B-cell activating factor
CD40-L: CD40 Ligand
