# Supplementary material for: CD71 expressing circulating neutrophils serve as a novel prognostic biomarker for metastatic spread and reduced outcome in pancreatic ductal adenocarcinoma patients
Source: Sci Rep. 2024 Sep 10;14:21164. doi: 10.1038/s41598-024-70916-3 (PMC11387421; doi:10.1038/s41598-024-70916-3)
Supplement: Supplementary file 1 — Supplementary Information. [file 41598_2024_70916_MOESM1_ESM.pdf]

Supplementary Figures

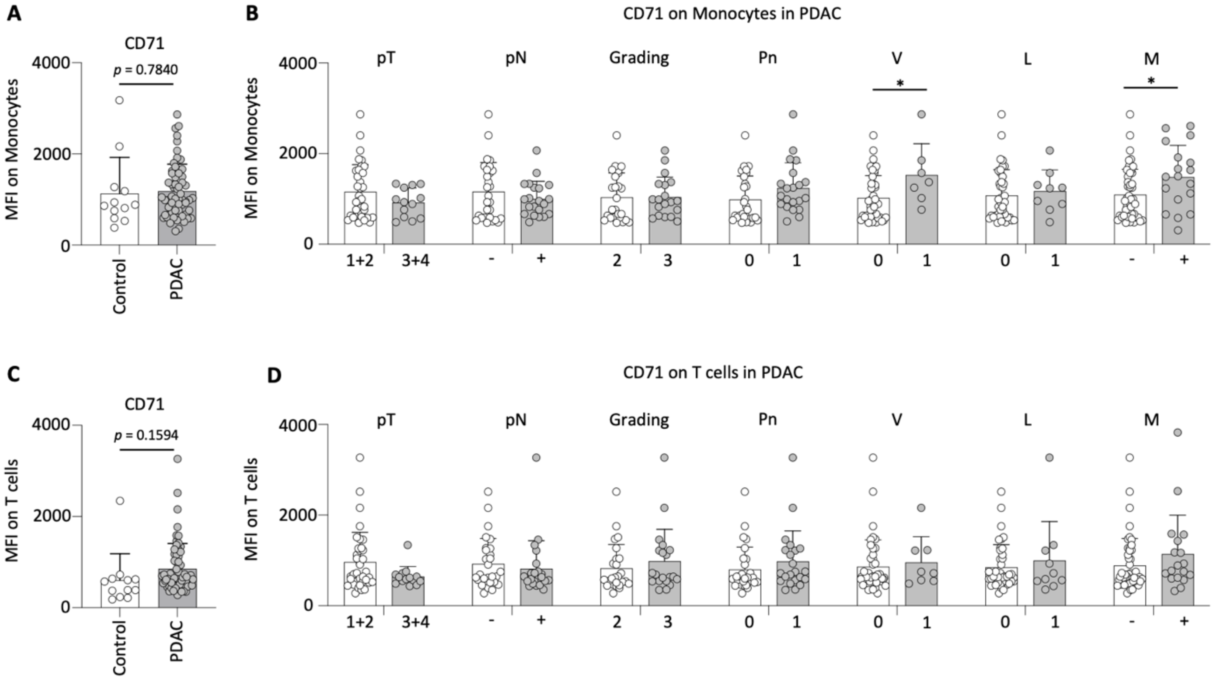

Supplementary Figure 1: The expression of CD71 on circulating Monocytes ($CD45^+HLADR^+CD14^+$) in clinical control (n = 12) and PDAC patients (n = 70) (A); expression of CD71 on circulating monocytes in PDAC patients correlated to the pT category (pT), pN category (pN), Grading, perineural invasion (Pn), Venous invasion (V), lymphatic invasion (L) and distant metastasis (M) (B); The expression of CD71 on circulating T cells ($CD45^+CD3^+$) in clinical control (n = 12) and PDAC patients (n = 70) (C); expression of CD71 on circulating T cells in PDAC patients correlated to the pT category (pT), pN category (pN), Grading, perineural invasion (Pn), Venous invasion (V), lymphatic invasion (L) and distant metastasis (M) (D); * $p < 0.05$.

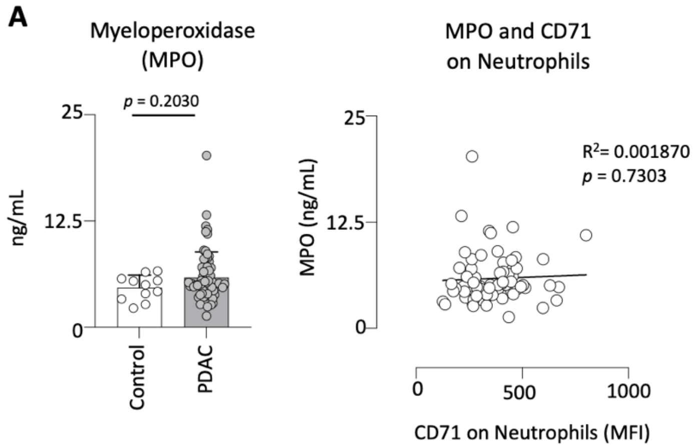

Supplementary Figure 2: Levels of the Myeloperoxidase (MPO) in the plasma of clinical control (n = 12) and PDAC patients (n = 70) and the correlation to the MFI of CD71 on circulating neutrophils.
